# Alkylimidazolium Ionic Liquids as Antifungal Alternatives: Antibiofilm Activity Against *Candida albicans* and Underlying Mechanism of Action

**DOI:** 10.3389/fmicb.2020.00730

**Published:** 2020-04-21

**Authors:** G. Kiran Kumar Reddy, Y. V. Nancharaiah

**Affiliations:** ^1^Biofouling and Biofilm Processes, Water and Steam Chemistry Division, Chemistry Group, Bhabha Atomic Research Centre, Kalpakkam, India; ^2^Homi Bhabha National Institute, Mumbai, India

**Keywords:** imidazolium ionic liquids, antifungal drugs, antibiofilm agents, *Candida albicans* biofilms, ergosterol content, membrane damage, mitochondrial dysfunction, oxidative stress

## Abstract

*Candida albicans* is an opportunistic pathogen causes fungal infections that range from common skin infections to persistent infections through biofilm formation on tissues, implants and life threatening systemic infections. New antifungal agents or therapeutic methods are desired due to high incidence of infections and emergence of drug-resistant strains. The present study aimed to evaluate (i) the antifungal and antibiofilm activity of 1-alklyl-3-methyl imidazolium ionic liquids ([CnMIM]^+^[X]^–^, *n* = 4, 12 and 16) against *Candida albicans* ATCC 10231 and two clinical *C. albicans* strains and (ii) the mechanism of action of promising antifungal ionic liquid on *C. albicans*. Two of the tested compounds were identified as more effective in preventing growth and biofilm formation. These ionic liquid compounds with –dodecyl and –hexadecyl alkyl groups effectively prevented biofilm formation by fluconazole resistant *C. albicans* 10231 and two other clinical *C. albicans* strains. Although both the compounds caused viability loss in mature *C. albicans* biofilms, an ionic liquid with –hexadecyl group ([C_16_MIM]^+^[Cl]^–^) was more effective in dispersing mature biofilms. This promising ionic liquid compound ([C_16_MIM]^+^[Cl]^–^) was chosen for determining the underlying mode of action on *C. albicans* cells. Light microscopy showed that ionic liquid treatment led to a significant reduction in cell volume and length. Increased cell membrane permeability in the ionic liquid treated *C. albicans* cells was evident in propidium iodide staining. Leakage of intracellular material was evident in terms of increased absorbance of supernatant and release of potassium and calcium ions into extracellular medium. A decrease in ergosterol content was evident when *C. albicans* cells were cultured in the presence of antifungal ionic liquid. 2′,7′-Dichlorodihydrofluorescein acetate assay revealed reactive oxygen species generation and accumulation in *C. albicans* cells upon treatment with antifungal ionic liquid. The effect of antifungal ionic liquid on mitochondria was evident by decreased membrane potential (measured by Rhodamine 123 assay) and loss of metabolic activity (measured by MTT assay). This study demonstrated that imidazolium ionic liquid compound exert antifungal and antibiofilm activity by affecting various cellular processes. Thus, imidazolium ionic liquids represent a promising antifungal treatment strategy in lieu of resistance development to common antifungal drugs.

## Introduction

Fungal pathogens are a major health issue causing over 1.6 million deaths annually (Almeida et al., 2019). Several species of *Candida* are responsible for the fungal infections, collectively called as candidiasis. These are commensal organisms in healthy individuals and reside in gastrointestinal, respiratory, and genitourinary tracts. In immunocompromised or diseased patients, they become opportunistic and cause infections ranging from superficial (oral or vaginal) to life threatening systemic infections^1^. About 50 to 70% of systemic fungal infections are caused by *Candida* spp. (Santos et al., 2018). *Candida albicans* is the most frequently observed organism in candidiasis. Persistent Candida infections are increasingly being reported in medically implanted devices such as catheters, heart valves, pacemakers, vascular bypass grafts, dentures and endotracheal tubes thus leading to high mortalities (Ramage et al., 2006; Sardi et al., 2013; Santos et al., 2018). Biofilm mode of growth by *Candida* spp. further complicates the treatment as the cells reside in biofilms are about 2000 times more resistant to fluconazole and amphotericin B over their planktonic counterparts (Bergamo et al., 2015).

Azoles, polyenes, allylamines, and echinocandins are the current antifungal arsenal available for treating candidiasis (Fuentefria et al., 2018). Fluconazole is most commonly used for treating candidiasis due to its low cost, high bioavailability and possibility of drug administration is various formulations (Martin, 1999). However, well documented resistance of *Candida* spp. to fluconazole makes this drug a less attractive antifungal agent in the current treatment scenario. Besides drug efflux mechanisms, alterations in target sites/gene expression, current challenges in treatment include biofilm formation which directly or indirectly enhances the drug resistance (Fuentefria et al., 2018). Currently applied strategies are ineffective against biofilms warranting prospective new antifungal agents from natural or synthetic origin (Sardi et al., 2013; Gyawali and Ibrahim, 2014; Nobile and Johnson, 2015). A potential antifungal agent should have broad spectrum activity in terms of antifungal and antibiofilm activities with minimal cytotoxicity and side effects to the host.

Ionic liquids are a novel class of molten salts at ≤100°C and exclusively made up of combination of cations and anions (Rogers and Seddon, 2003). These salts typically comprises of a large cationic core (often a nitrogen containing group with alkyl substituent) and a small counter anion (Pendleton and Gilmore, 2015). Apart from their applications in chemical industry (Vekariya, 2017), these compounds are promising as components of active pharmaceutical ingredients and antimicrobials (Egorova et al., 2017). The tunable property of ionic liquids by way of changing their constituent ions, allows making large structural diversity of about 10^18^ compounds (Pernak et al., 2007) with altered physical, chemical and biological activities. Some of these ionic liquids have been explored as antimicrobials, antiseptics and antifouling agents (Pernak et al., 2004; Nancharaiah et al., 2012; Egorova et al., 2017). Imidazolium ionic liquids have been reported for effective control of bacterial and phototrophic biofilms (Carson et al., 2009; Busetti et al., 2010; Reddy et al., 2017, 2020). With respect to antifungal activity, –ethyl and –butyl side chain containing imidazolium, pyridinium and cholinium ionic liquids were evaluated against *Penicillium* sp. (Petkovic et al., 2009). Schrekker et al. (2013) reported the efficient antifungal activity of N-alkyl-substituted imidazolium salts with –decane, –tetradecane and –hexadecane side chain containing cations against fungal pathogens with minimum toxicity to leukocytes. Activity of –hexadecyl side chain containing imidazolium ionic liquid against multidrug resistant *Candida tropicalis* and clinical dermatophyte strains showed potential activity against biofilms (Bergamo et al., 2014, 2015; Dalla Lana et al., 2015). Inhibition of conidia germination and mycelial growth was observed in *Fusarium graminearum* (Ribas et al., 2016). Antifungal ionic liquids were incorporated into poly(L-lactide) biomaterials for inhibiting adhesion of *Candida* spp. (Schrekker et al., 2016). Using contaminated acrylic resin strip specimens, 1-n-hexadecyl-3-methylimidazolium chloride was demonstrated as a strong antifungal for mouthwash formulation (Bergamo et al., 2016). Although several studies have reported antifungal activity, effect of imidazolium ionic liquids on preformed fungal biofilms (biofilm eradication potential) is largely unknown (Table 1). Evaluation of antifungal ionic liquids on preformed biofilms is of clinical relevance as the biofilm formation often precedes treatment. Cell membrane has been identified as the potential target in the case of imidazolium ionic liquids (Nancharaiah et al., 2012; Benedetto, 2017; Egorova et al., 2017). Other studies indicated that imidazolium ionic liquids can decrease the content of ergosterol, an important component of fungal cell membranes (Schrekker et al., 2013). Ionic liquids are currently seen as promising asset for fighting fungal infections (Hartmann et al., 2016). Due to limited understanding of mechanisms, studies aimed at identifying potential targets and underlying mode of action of antifungal ionic liquids are desired for their prospective use in treating infections.

**TABLE 1:** Summary of studies on evaluation of ionic liquids against various fungal strains.

Ionic liquid	Fungi	MIC (μmol l^–1^)	MFC (μmol l^–1^)	Effective Antibiofilm concentrations	Biofilm eradication	Reference
[C_16_MIM]Cl	6 isolates of *C. tropicalis*	0.04	ND	0.08–0.65	Killing of biofilm cells.	Bergamo et al., 2014
[C_n_MIM]Cl (*n* = 4, 10, 12, 16, 18);	21 strains of *Microsporum* sp.; 24 strains of *Trichophyton* sp.	Avg MIC_50_ C_4_ = 71; C_10_ = 10.16; C_12_ = 6.59; C_16_ = 0.20; C_18_ = 20.03.	C_16_ = 0.6–36 for *Microsporum* sp.; C_16_ = 0.23–36.4 for *Trichophyton* sp.	ND	ND	Dalla Lana et al., 2015
[C_n_MIM]MeS (*n* = 4, 9, 16)	21 strains of *Microsporum* sp.; 24 strains of *Trichophyton* sp.	Avg MIC_50_ C_4_ = 53; C_9_ = 17.37 C_16_ = 0.12.	C16 = 0.5–31 for *Microsporum* sp.; C16 = 0.5–31 for *Trichophyton* sp.	ND	ND	Dalla Lana et al., 2015
[C_16_MIM]Cl; [C_16_MIM]MeS	*Candida tropicalis*	ND	ND	ND	Killing of biofilm cells	Bergamo et al., 2015
[C_16_MIM]Cl; [C_16_MIM]MeS	4 *Fusarium graminearum* strains	9.1–18.2 and 7.7–15.5	ND	ND	ND	Ribas et al., 2016
[C_n_MIM]Cl (*n* = 4, 12, 16)	Three fluconazole resistant *C. albicans* strains	C_4_ ≥ 1000, C_12_ = 25, C_16_ = 4.68	C_4_ ≥ 1000, C_12_ = 75, C_16_ = 6.25	C_4_ = NE, C_12_ = 25, C_16_ = 6.25	C_4_ = NE; C_12_ = Biofilm cell killing; C_16_ = Killing and biofilm removal	Current study

*[C_n_MIM]Cl: 1-n-Alkyl-3-methylimidazolium chloride; [C_n_MIM]MeS: 1-n-Alkyl-3-methylimidazolium methanesulfonate; ND, not determined; NE, no effect.*

This study was aimed to determine the antifungal, antibiofilm and biofilm eradication activities of three imidazolium ionic liquids against *C. albicans* strains and to understand the mode of action of potent antifungal imidazolium ionic liquid. Antibiofilm activity was determined as prevention of biofilm formation in the presence of ionic liquids and antifungal drugs. Biofilm eradication was determined in terms of killing and dispersal activity of ionic liquids on preformed fungal biofilms.

## Materials and Methods

### Organisms, Media and Growth Conditions

This research was conducted using *C. albicans* ATCC 10231 (Microbiologics, United States), a reference strain commonly used for evaluating antifungal and antibiofilm agents. Experiments were conducted with two clinical strains of *C. albicans* [CA i16 (GenBank No. MG757722.1) and CA i21 (GenBank No. MG757724.1)] which were isolated from sputum samples of patients. The clinical strains were obtained from University of Madras, India. These cultures were routinely maintained on potato dextrose agar (PDA) (HiMedia, India). For liquid cultures, a single colony was picked from PDA, transferred to potato dextrose broth (PDB) and incubated for 24 h at 30°C and 120 RPM in a temperature controlled orbital shaker. Cells harvested from PDB were used for growth and biofilm experiments.

Filter sterilized RPMI 1640 medium (L-Glutamine, phenol red, 2 g l^–1^ glucose and 0.165 mol l^–1^ MOPS buffer, pH 7.0) (Part No. AT180, HiMedia, India) was used for biofilm experiments. Cultures were grown in PDB for 24 h, pelleted by centrifugation, re-suspended in RPMI 1640 and adjusted to desired cell density for performing biofilm experiments. For determining the mechanism of action, cells were re-suspended in phosphate buffered saline (PBS).

### Imidazolium Ionic Liquids and Antifungal Drugs

1-butyl-3-methylimidazolium chloride ([C_4_MIM][Cl]), 1-dodecyl-3-methylimidazolium iodide ([C_12_MIM][I]), fluconazole and amphotericin B were purchased from Sigma-Aldrich (United States). 1-hexadecyl-3-methylimidazolium chloride ([C_16_MIM][Cl]) was purchased from Acros (United States). The chemical structures of three ionic liquids used in this study are given in Supplementary Information (Supplementary Figure S1). Stock solutions (100 mmol l^–1^) of ionic liquids were prepared in sterile, ultrapure water and stored at room temperature until further use. Stock solutions of fluconazole (32 mmol l^–1^) and amphotericin B (13 mmol l^–1^) were prepared, respectively, in ethanol and dimethyl sulfoxide (DMSO). These stock solutions were stored at 4°C until use.

### MIC and MFC of Ionic Liquids Against *C. albicans* ATCC 10231

MIC of ionic liquids was determined by microdilution method according to the Clinical and Laboratory Standards Institute (CLSI) guidelines [CLSI (2012)]. Actively growing log phase culture in PDB was pelleted by centrifugation (8000 rpm for 5 min). Cell pellet was re-suspended in RPMI 1640 and cell density was adjusted. MIC and MFC values were determined in RPMI 1640 medium containing initial cell densities of 10^3^ or 10^6^ cfu ml^–1^. Initial cell density of 10^3^ cfu ml^–1^ is recommended for determining MIC according to CLSI antifungal susceptibility testing [CLSI (2012)]. Initial cell density of 10^6^ cfm ml^–1^ often employed in biofilm inhibition studies was also used for determining MIC and MFC values according to Pierce et al. (2008). A two-fold series dilution of ionic liquids and antifungal drugs (fluconazole and amphotericin B) were prepared in RPMI 1640 containing *C. albicans* cells. Working concentrations in the range of 1000 to 31.25 μmol l^–1^ for [C_4_MIM][Cl], 100 to 1.15 μmol l^–1^ for [C_12_MIM][I] and 50 to 1.15 μmol l^–1^ for [C_16_MIM][Cl] were prepared in RPMI 1640 and tested. Concentrations of fluconazole and amphotericin B were, respectively, in the range of 3265 to 12.75 μmol l^–1^ and 13 to 0.16 μmol l^–1^. Subsequently, 200 μl of these dilutions were transferred to the wells of 96-well microtiter plate. Five replicates were set up for each concentration. Control wells received cells in RPMI without the compound. After 24 h of incubation in an orbital shaker at 37°C and 120 RPM, growth was determined by measuring absorbance at 600 nm using a microplate reader (BioTek^®^, United States). The lowest concentration that prevented *C. albicans* growth (measured as absorbance at 600 nm) was represented as MIC. For MFC estimation, aliquots of suspension from selected microtiter wells were plated onto PDA plates and incubated at 37°C for 48 h. MFC was the lowest concentration at which no colonies of *C. albicans* appeared on PDA plates.

### Antibiofilm Activity of Ionic Liquids

For estimating incubation time for maximum biofilm formation, RPMI 1640 containing 10^6^ cfu ml^–1^ was aliquoted (200 μl) in 96-well sterile, flat bottom, polystyrene microtiter plates (Tarsons, India). Pierce et al. (2008) recommended 10^6^ cfu ml^–1^ as inoculum cell density for developing *C. albicans* biofilms in 96-well plates. Hence, 10^6^ cfu ml^–1^ cells were used for all biofilm experiments. Multi-well plates containing cells in RPMI 1640 were incubated at 120 RPM and 37°C for 6, 12, 18, 24 or 48 h. At the end of incubation, biofilm mass and metabolic activity were estimated by crystal violet (CV) and 2,3-Bis-(2-Methoxy-4-Nitro-5-Sulfophenyl)-2H-Tetrazolium-5-Carboxanilide (XTT), respectively. Based on time course experiment on biofilm formation, 24 h incubation time was chosen for quantifying the effect of ionic liquids and antifungal drugs on biofilm formation.

Antibiofilm activity was determined by incubating planktonic and adherent cells separately in the presence of ionic liquids and antifungal drugs. *C. albicans* cell suspensions (200 μl, 10^6^ CFU/ml in RPMI 1640) were transferred to each well of a 96-well microtiter plate. Ionic liquids and antifungal drugs were added and serially diluted using two-fold dilution. Final concentrations in the range of 1000 to 125 μmol l^–1^ for [C_4_MIM][Cl], 50 to 2.3 μmol l^–1^ for [C_12_MIM][I] and 25 to 1.1 μmol l^–1^ for [C_16_MIM][Cl] were tested. Amphotericin B was tested in the range of 13 to 0.168 μmol l^–1^. Fluconazole concentrations were tested up to 3265 μmol l^–1^. Effect of anions (Cl^–^ and I^–^) and 1-methylimidazole on growth and biofilm formation of *C. albicans* was determined by incubating with excess concentrations (500–1000 μmol l^–1^) of NaCl, KI and 1-methylimidazole. For each concentration, five replicate wells were used. The plates were incubated at 37°C in an orbital shaker at 120 rpm to allow biofilm formation. For determining antibiofilm activity using adherent cells, the cell suspensions were transferred to each well of a microtiter plate and incubated for 3 h at 37°C in an orbital shaker at 120 rpm to allow adhesion. Subsequent to adhesion, the non-attached cells were carefully removed from the wells. Then 200 μl of RPMI medium containing different concentration of ionic liquids and antifungal drugs was added to each well. The plates were incubated for 24 h as described above to allow biofilm formation.

The biofilm was quantified using the both CV assay (Nancharaiah et al., 2012) and XTT reduction assay (Henriques et al., 2006). For CV assay, biofilms were stained with 0.1% CV (HiMedia, India) for 10 min. Excess CV was removed by washing the wells with demineralized water (Nancharaiah et al., 2012). Plates were air-dried for overnight and CV bound to the biofilm was eluted with 33% glacial acetic acid. Eluted CV was measured by reading absorbance at 570 nm. Eluted CV was diluted with 33% glacial acetic acid whenever the absorbance exceeded 2. For XTT reduction assay, working solutions of XTT (0.5 mg ml^–1^) were prepared in sterile PBS, stored as 1.8 ml aliquots at −18°C. As an electron coupler, a stock solution of 0.32 mg ml^–1^ phenazine methosulfate (PMS) was prepared in PBS and stored at −18°C in 0.2 ml aliquots. Prior to the assay, 1.8 ml of XTT was mixed with 0.2 ml PMS and added immediately to each well of the microtiter plate. The plates were incubated at 37°C for 2 h to develop orange colored formazan which was estimated at 492 nm (Nett et al., 2011).

For visualization of biofilms, sterile glass slides were inserted in 50 ml falcon tubes containing 25 ml of RPMI 1640 with 10^6^ cfu ml^–1^ cells and different concentrations of ionic liquids. Tubes were incubated at 37°C, 120 RPM for 24 h. Slides were washed with PBS to remove loosely bound cells, stained with BacLight^®^ live/dead stain (Invitrogen, United States) for 15 min and observed under inverted fluorescence microscope (Carl Zeiss, Germany).

### Eradication of Preformed Biofilms by Ionic Liquids

For biofilm eradication experiments, 24 h old biofilms were cultivated in RPMI 1640 as described above. After 24 h, spent media was discarded from wells and washed with PBS buffer to remove loosely bound *C. albicans* cells. Different concentrations of ionic liquids were prepared by two-fold dilution in PBS and 200 μl aliquots were transferred to wells. Five wells were used for each concentration. Plates were incubated again for 24 h at 37°C and 120 RPM. After the challenge period, contents in wells were discarded and washed with PBS to remove detached or loosely bound cells. The biofilm remained after exposure to test compounds was quantified with CV assay. Viability and metabolic activity of cells in the challenged biofilms was determined using BacLight^®^ staining and XTT reduction, respectively. For this, working solution of Syto 9 and propidium iodide (PI) mixture was prepared as per manufacturer’s recommendations. Challenged biofilms were stained with 200 μl of stain in the dark for 15 min. Syto 9 and PI fluorescence was estimated by multimode reader (BioTek^®^, United States) using 488 nm excitation. Syto 9 and PI signals were collected at 520 and 620 nm, respectively. The results were represented as the ratio of Syto 9 to PI fluorescence (Live/Dead ratio). XTT reduction assay was performed as mentioned previously.

### Effect of Ionic Liquids on Clinical *C. albicans* Isolates

Biofilm forming clinical isolates (CA i16 and CA i21) were screened for evaluating the efficacy of fluconazole, amphotericin B and ionic liquids. These isolates were cultured overnight in PDB and adjusted to a cell number of 10^6^ cfu ml^–1^ in RPMI 1640. Biofilm formation by CA i16 and CA i21 was determined at different time intervals as mentioned above. Then antibiofilm and biofilm eradication experiments were performed in the presence of ionic liquids. Similar experimental procedures, incubation time and estimation assays were used, as mentioned previously.

### Mechanism of Action of Antifungal Ionic Liquids

Based on MIC, MFC, antibiofilm and biofilm eradication against *C. albicans* and clinical isolates, the potential ionic liquid [C_16_MIM][Cl] was selected for identifying possible mode of action. From the previously estimated MIC values, a 10-fold MIC concentration of [C_16_MIM][Cl] and amphotericin B were prepared in sterile PBS and incubated with 10^6^ cfu ml^–1^ for 3 h at 120 RPM. At the end of incubation, cells exposed to the ionic liquid were harvested by centrifugation, washed with PBS and used for various assays (morphological changes, cell membrane permeabilization, leakage of intracellular material, reactive oxygen species and mitochondrial dysfunction) described below. For determining effect on ergosterol content, *C. albicans* 10231 was grown in PDB with sub-MIC concentrations of antifungal ionic liquid.

### Morphological Changes Upon Ionic Liquid Exposure

*Candida albicans* cells exposed to [C_16_MIM][Cl] were harvested and observed under bright field microscope. The images were analyzed using *ImageJ* 1.37V software for determining the size of cells in terms of overall length. For each treatment, a minimum of 220 cells were analyzed and average cell length was determined.

### Effect on Membrane Permeabilization

Alteration in cell membrane permeability was determined by investigating the propidium iodide (PI) uptake by *C. albicans* cells. *C. albicans* cells exposed to ionic liquid were harvested by centrifugation, washed with PBS and stained with 20 μM PI (Invitrogen, United States) for 15 min. Experiment was performed in triplicates. PBS was used as the control. Inverted fluorescence microscope (Carl Zeiss, Germany) was used for visualizing cells exhibiting PI fluorescence. PI uptake by the *C. albicans* cells was measured quantitatively using 485 nm excitation and 630 nm emission settings with multimode reader (BioTek^®^, United States).

### Leakage of Intracellular Material

Leakage of intracellular contents was indirectly measured by the increase in concentrations of metal cations such as potassium and calcium and increase in 260 nm absorbance in cell free supernatants. *C. albicans* cells (10^6^ cfu ml^–1^) in ultra-high pure (UHP) water was exposed to 0.1, 0.25, 0.5, 0.75, and 1 mM of [C_16_MIM][Cl]. After 3 h incubation at 37°C and 120 RPM, cell suspensions were centrifuged to collect the supernatant. Absorbance of the supernatant was measured at 260 nm using UV-Visible spectrophotometer (Shimadzu). Metal cations were measured in the supernatants by inductively coupled plasma-atomic emission spectrometer (ICP-AES) (Horiba Jovin Yvon, France). Appropriate controls (UHP water, UHP water with 0.1 to 1 mM [C_16_MIM][Cl]) were used for analyzing absorbance at 260 nm and quantifying metal cations.

### Effect on Ergosterol Content

For this, *C. albicans* cells were cultured in 50 mL Erlenmeyer flasks containing 20 ml PDB with sub-MIC concentrations (MIC/2, MIC/4 and MIC/8) of [C_16_MIM][Cl] and fluconazole (5 and 50 μM). Flasks were incubated for 24 h at 120 RPM and 37°C. Then, cells were harvested, washed with PBS and used for extracting total sterols through saponification (Arthington-Skaggs et al., 2000). Briefly, 3 mL of 25% alcoholic KOH solution (25 g KOH dissolved in 36 ml UHP water with a 100 mL final make up with 100% ethanol) was added to each of the cell pellet in falcon tubes. Each of these suspensions were mixed by vortexing for a minute and incubated for 1 h in 80°C water bath. After incubation, tubes were cooled to room temperature and sterols were extracted by adding a mixture of water (1 mL) and n-hexane (3 mL). These suspensions were vigorously mixed by vortexing for 3 min and left for phase separation. The hexane layer containing sterols was diluted with absolute ethanol and scanned between 200 and 300 nm using UV-Vis spectrophotometer. Ergosterol content was determined and normalized to wet biomass content using established formulae (Arthington-Skaggs et al., 2000).

### Effect on Intracellular Reactive Oxygen Species (ROS)

*Candida albicans* cells exposed to [C_16_MIM][Cl] were analyzed for intracellular ROS using 2’,7’-Dichlorodihydrofluorescein diacetate (DCFH-DA) (Sigma-Aldrich, United States) (Kobayashi et al., 2002). Cell number, treatment with [C_16_MIM][Cl], incubation and washing procedures were similar to that of membrane permeabilization experiment. Cells exposed to ionic liquid were incubated with 20 μM DCFH-DA at 37°C under dark for 30 min. Experiment was performed in four replicates with appropriate controls. The cells were then observed for 2’,7’-dichlorofluorescein (DCF) fluorescence using an inverted fluorescence microscope (Carl Zeiss, Germany). The fluorescence of DCF was quantified with 485 nm excitation and 538 nm emission settings using multimode reader (BioTek^®^, United States).

### Effect on Mitochondrial Membrane Potential and Mitochondrial Activity

Mitochondrial membrane potential (Δψ_m_) in *C. albicans* after exposure to [C_16_MIM][Cl] was measured using Rhodamine 123 (Rh123) (Sigma, United States) as per Lopes et al. (2013) with minor modifications. Cells were exposed to [C_16_MIM][Cl] as detailed in the membrane permeabilization experiment. Experiment was conducted in four replicates. The cells exposed to ionic liquid were stained with 20 μM Rh123 for 30 min in the dark. After incubation, excess stain was removed and washed with PBS. Cells were re-suspended in PBS and observed for Rh123 fluorescence under inverted fluorescence microscope (Carl Zeiss, Germany) through FITC filter. Fluorescence of Rh123 was also quantified with 485 nm excitation and 530 nm emission settings using multimode reader (BioTek^®^, United States).

MTT assay was used for determining mitochondrial activity of *C. albicans* cells (Lopes et al., 2013). Cells exposed to [C_16_MIM][Cl] were harvested, re-suspended in PBS containing 0.5 mg ml^–1^ MTT. Cells were incubated at 120 RPM and 37°C for 2 h. End of the incubation, cell suspensions were centrifuged and washed with PBS. Purple formazan product developed from the MTT reduction by mitochondrial dehydrogenases was solubilized in 200 μL DMSO by vigorous vortexing. Suspensions were centrifuged and supernatants were collected for estimating formazan absorbance at 510 nm using UV-Visible spectrophotometer (Shimadzu, Japan). Experiment was performed in duplicates with necessary controls.

### Statistical Analysis

Data was processed from replicates and presented as mean ± standard deviation (SD). Student’s *t*-test was used for determining the statistical significance. Differences between the control and treatment samples were considered to be significant at *P*-values <0.05, <0.01, and <0.001.

## Results

### MIC and MFC of Ionic Liquids and Antifungal Drugs

The antifungal activity of three ionic liquids and antifungal drugs was expressed as MIC and MFC against *C. albicans* 10231 (Table 2). Growth of *C. albicans* 10231 was not inhibited in the presence of [C_4_MIM][Cl] even at the highest concentration (1000 μmol l^–1^) tested using initial cell densities of 10^3^ and 10^6^ cfu ml^–1^. Thus, growth of *C. albicans* 10231 in the presence of [C_4_MIM][Cl] was similar to that of control. However, the growth of *C. albicans* 10231 was severely inhibited in the presence of imidazolium ionic liquids containing –dodecyl or –hexadecyl alkyl groups. [C_12_MIM][I] completely inhibited the growth of 10^3^ and 10^6^ cfu ml^–1^ cell densities, respectively, at 6.25 and 25 μmol l^–1^. MFC values at these cell densities was determined to be 37.5 and 75 μmol l^–1^, respectively. Among the tested compounds, [C_16_MIM][Cl] showed maximum potency with MIC value of 2.34 and 4.68 μmol l^–1^, for 10^3^ and 10^6^ cfu ml^–1^, respectively. The MFC concentrations for [C_16_MIM][Cl] were determined to be 4.68 and 6.25 μmol l^–1^, for 10^3^ and 10^6^ cfu ml^–1^, respectively. The MIC values for fluconazole and amphotericin B were determined to >3265 μmol l^–1^ and 1.62 μmol l^–1^ against 10^6^ cfu ml^–1^ *C. albicans* 10231, respectively. The MFC values showed that [C_16_MIM][Cl] was more effective in killing fungal cells than other two ionic liquids and fluconazole. The MFC of [C_16_MIM][Cl] at 6.25 μmol l^–1^ was slightly greater as compared to 1.62 μmol l^–1^ for amphotericin B.

**TABLE 2:** Antifungal activities of imidazolium ionic liquids and antifungal drugs against *Candida albicans* reference strain (*C. albicans* 10231) and clinical strains (CA i16 and CA i21).

Strain*	Compound (μmol l^–1^)									
	[C_4_MIM][Cl]		[C_12_MIM][I]		[C_16_MIM][Cl]		Fluconazole		Amphotericin B	
	MIC	MFC	MIC	MFC	MIC	MFC	MIC	MFC	MIC	MFC
*C. albicans* 10231	>1000	ND	25	75	4.68	6.25	>3265	ND	1.62	1.62
CA i16	>1000	ND	25	50	4.68	6.25	>3265	ND	0.34	0.68
CA i21	>1000	ND	37.5	75	9.38	12.5	>3265	ND	0.67	1.34

*Initial cell density: 10^6^ cfu ml^–1^. ND, not determined.

### Antibiofilm Activity of Ionic Liquids

**FIGURE 1:**
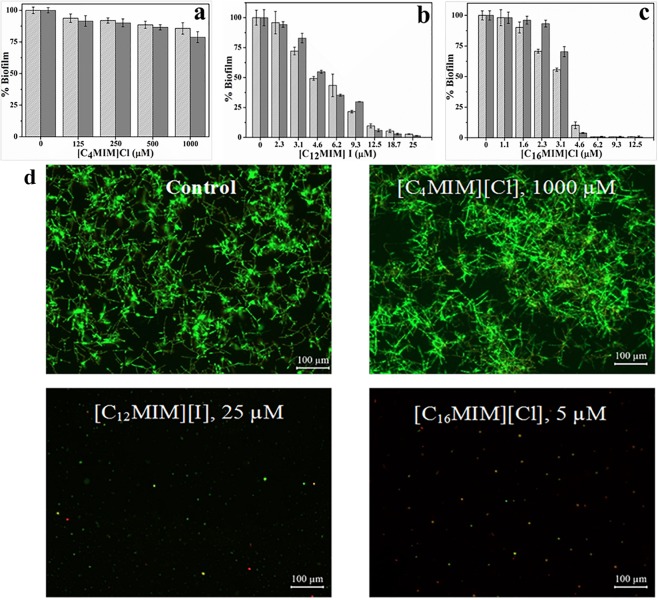
Biofilm formation by *C. albicans* 10231 at different initial concentrations of [C_4_MIM][Cl] **(a)**, [C_12_MIM][I] **(b)**, and [C_16_MIM][Cl] **(c)**. Biofilm quantified by CV (light gray) and XTT (dark gray) was presented. Fluorescence microscopic images of *C. albicans* biofilms in presence of different concentrations of ionic liquids **(d)**.

Time course assay of biofilm development by *C. albicans* 10231 revealed highest biofilm mass and metabolic activity at 24 h (Supplementary Figure S2). Thus, 24 h incubation time was used for biofilm inhibition experiments. Biofilm formed in presence of different concentrations of imidazolium ionic liquids was quantified by CV and XTT as shown in Figure 1. Biofilm inhibition was not evident in the presence of [C_4_MIM][Cl] (Figures 1a,d). Biofilm formation by *C. albicans* 10231 was severely inhibited in the presence of imidazolium ionic liquids containing –dodecyl and –hexadecyl groups. For example, 50 and 100% inhibition in biofilm formation was achieved, respectively, using 4.6 and 25 μmol l^–1^ [C_12_MIM][I] (Figure 1b). [C_16_MIM][Cl] was found to be more potent with no biofilm formation beyond 4.68 μmol l^–1^ (Figure 1c). Biofilm formation with adhesion step revealed complete inhibition in biofilm formation beyond 25 and 6.25 μmol l^–1^ for [C_12_MIM][I] and [C_16_MIM][Cl], respectively (Supplementary Figure S3). Complete inhibition in biofilm formation was achieved using 1.62 μmol l^–1^ amphotericin B. However, biofilm formation was not prevented even using 3265 μmol l^–1^ of fluconazole. Inhibition of *C. albicans* 10231 biofilm formation in the presence of imidazolium ionic liquids with –dodecyl or –hexadecyl groups was clearly evident in the florescence microscopic images (Figure 1d). The anion constituents (Cl^–^ and I^–^) and 1-methylimidazole ring did not show any significant effect on the growth and biofilm formation of *C. albicans* 10231 (Supplementary Figure S4).

### Biofilm Eradication Potential of Ionic Liquids

**FIGURE 2:**
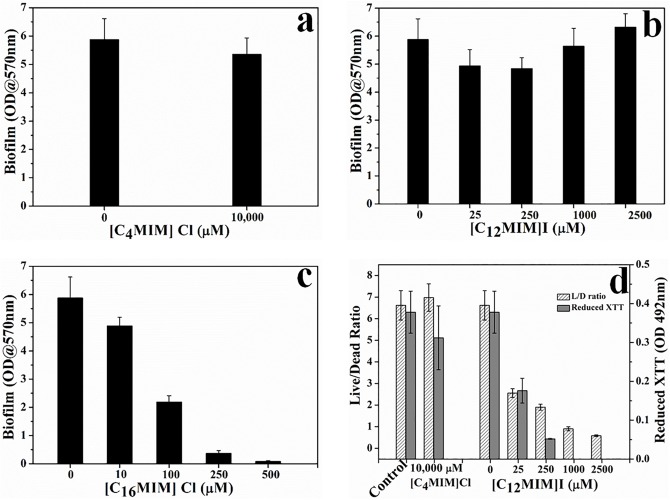
Effect of imidazolium ionic liquids on preformed biofilms. *C. albicans* 10231 biofilms were exposed to imidazolium ionic liquids for 24 h and then residual biofilm was quantified using CV method **(a–c)**. Cell viability in *C. albicans* biofilms upon exposure to ionic liquids that did not exert biofilm dispersal **(d)**.

The biofilm eradication potential of alkylimidazolium ionic liquids was determined in terms of killing of biofilm cells and dislodgement of preformed biofilm. The effect of alkylimidazolium ionic liquids on preformed *C. albicans* 10231 was shown in Figures 2 and Supplementary Figure S5. [C_4_MIM][Cl] and [C_12_MIM][I] did not cause dispersal of 24 h old *C. albicans* 10231 biofilms irrespective of their concentrations (Figures 2a,b). A concentration dependent biofilm dispersal was observed in the case of [C_16_MIM][Cl]. However, biofilm dispersal required much higher concentrations than those required for inhibiting biofilm formation. For example, removal of >90% of biofilm was required 250 μmol l^–1^ [C_16_MIM][Cl] (Figure 2c).

Since [C_4_MIM][Cl] and [C_12_MIM][I] were not causing any biofilm dispersal, their effect was determined in terms of viability and metabolic activity of biofilm cells (Figure 2d). There was no change in the Live/Dead (L/D) status or metabolic status of *C. albicans* 10231 biofilms exposed to 1000 μmol l^–1^ [C_4_MIM][Cl] for 24 h. However, the L/D status and XTT reduction potential of *C. albicans* 10231 biofilms was drastically decreased upon exposure to 25 to 2500 μmol l^–1^ of [C_12_MIM][I].

### Ionic Liquids Against Fluconazole Resistant Clinical *C. albicans* Strains

Clinical strains (CA i16 and CA i21) were selected based on their copious biofilm formation potential and high resistance toward fluconazole. CLSI has fixed MIC breakpoint of ≥64 μg ml^–1^ for fluconazole resistant strains (Manoharan et al., 2017). The clinical strains evaluated in this study were highly resistant to fluconazole and inhibition in growth was not observed up to 1000 μg ml^–1^ fluconazole. MIC and MFC values for ionic liquids and antifungal drugs against clinical *C. albicans* strains were presented in Table 2. Biofilm formation potential and metabolic activity of both the clinical strains at different time intervals was shown in Supplementary Figure S6. Biofilm formation pattern was found to be very similar between the two clinical strains. Incubation time of 24 h was considered optimum based on highest metabolic activity. The tested imidazolium ionic liquids with –dodecyl and –hexadecyl groups were effective in preventing biofilm formation by both the clinical strains. However, there was a marginal difference in the activity toward these two different isolates. Complete inhibition in biofilm formation by clinical isolates required 25 and 6.25 μmol l^–1^, respectively, for [C_12_MIM][Cl] and [C_16_MIM][I] (Supplementary Figure S7). Imidazolium ionic liquid with –hexadecyl group was equally effective in dispersing preformed biofilms of clinical strains. Removal of preformed biofilms of clinical strains was about 75 and 100% at 100 and 250 μmol l^–1^, respectively.

### [C_16_MIM][Cl] Causes Shrinking of *C. albicans* Cells

**FIGURE 3:**
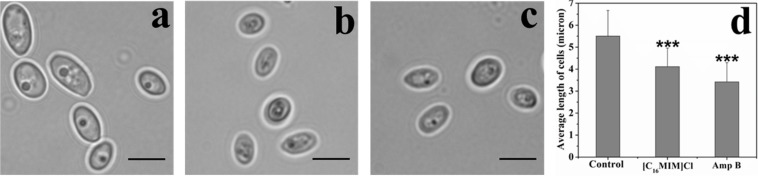
Effect of imidazolium ionic liquids on cell size during short term exposure. Bright field microscopic images of *C. albicans* cells in control **(a)**, [C_16_MIM][Cl] **(b)** and amphotericin B **(c)** treatments. Scale bar = 5 μm. Graph **(d)** represents the average length of cells in control and treatment. Cells exposed to 10× MIC concentration for 3 h; ***P < 0.001, *n* = 220.

Initial microscopy observations revealed that potent ionic liquids (containing -dodecyl, -hexadecyl side chain) cause changes in cell size (data not shown). A systematic study with [C_16_MIM][Cl] exposure to *C. albicans* 10231 cells revealed a significant decrease in the cell size (Figure 3). In control population, the cell length was 5.5 ± 1.1 μm. However, the cell length decreased to 4.1 ± 0.8 μm upon exposure to [C_16_MIM][Cl]. In presence of amphotericin B, the average length of cells was further reduced to 3.4 ± 0.8 μm (Figure 3d). The results indicate that, [C_16_MIM][Cl] decreased the cell volume leading to shrinkage of *C. albicans* 10231 cells.

### [C_16_MIM][Cl] Induces Membrane Permeabilization

**FIGURE 4:**
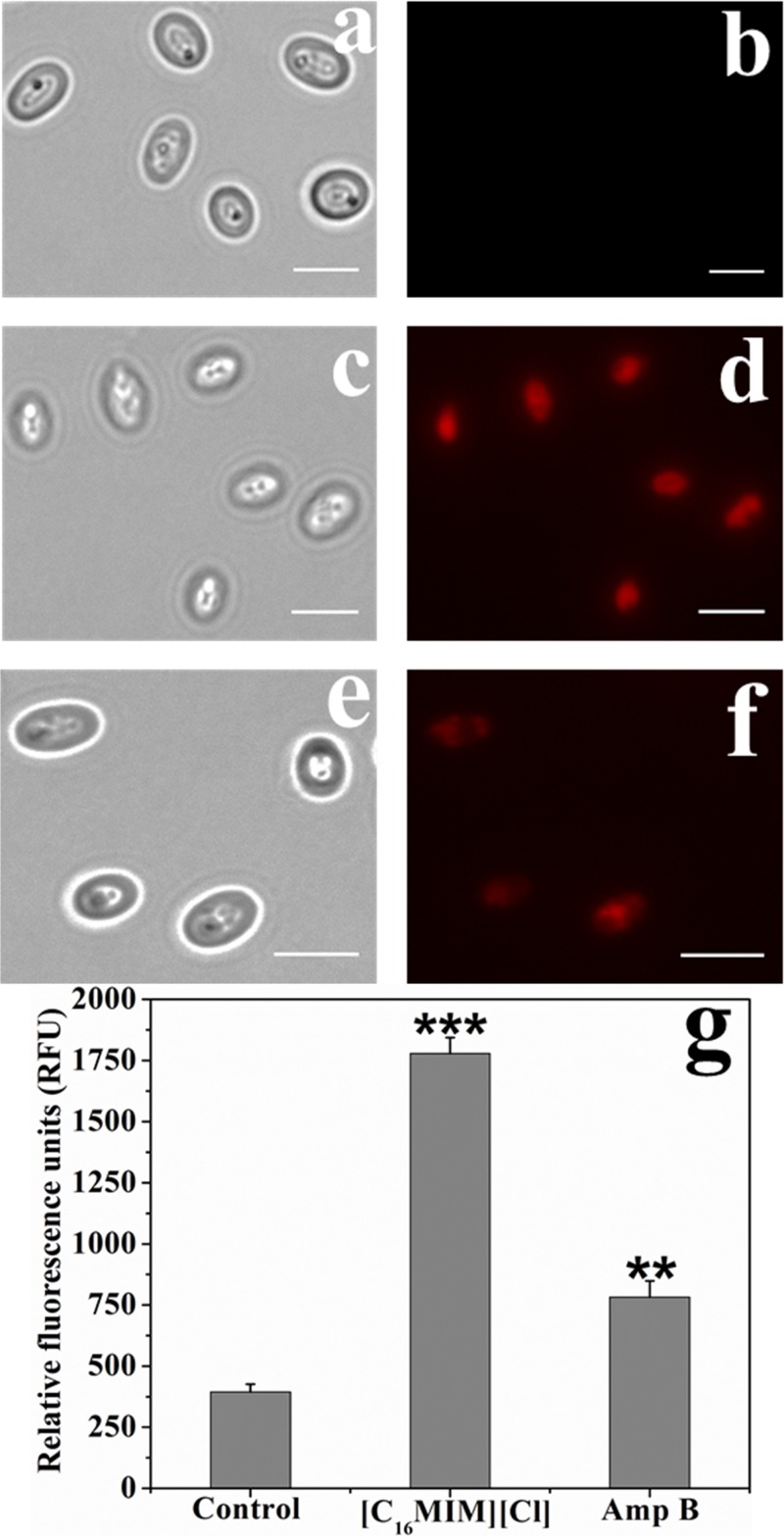
Propidium iodide (PI) uptake by *C. albicans* cells exposed to [C_16_MIM][Cl]. Bright field and fluorescence microscopy images of control **(a,b)**, ionic liquid treated **(c,d)** and amphotericin B treated **(e,f)** cells. **(g)** PI fluorescence by control, ionic liquid and amphotericin B treated *C. albicans* cells (cells exposed to 10× MIC concentration for 3 h; ***P < 0.001, **P < 0.01, *n* = 3).

Impact of [C_16_MIM][Cl] on plasma membrane was monitored by PI uptake. Due to high molecular weight, PI can only enter the cells with permeabilized cell membrane. Control cells were not stained by PI (Figure 4b). But, ionic liquid and amphotericin B treated cells were stained by the PI (Figure 4d,f). Quantitative fluorescence measurement revealed significant uptake of PI by the cells exposed to [C_16_MIM][Cl] than amphotericin B (Figure 4g).

### [C_16_MIM][Cl] Causes Leakage of Intracellular Material

**FIGURE 5:**
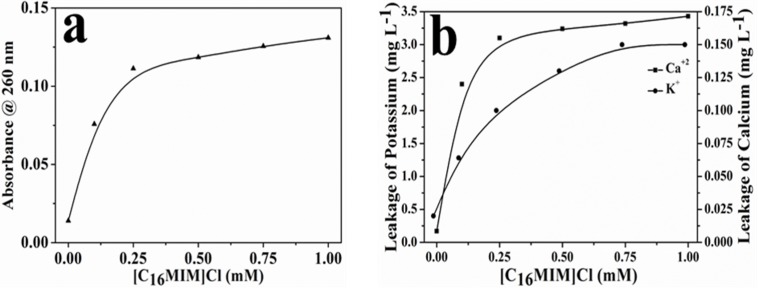
Leakage of intracellular components from *C. albicans* cells upon exposure to [C_16_MIM][Cl]. **(a)** Absorbance of cell-free supernatant at 260 nm, **(b)** concentration of potassium and calcium in cell-free supernatant.

[C_16_MIM][Cl] induced leakage of intracellular contents was measured from the increase in 260 nm absorbance (nucleic acids) and release of important metal cations (i.e., potassium and calcium). A clear increase in the absorbance at 260 nm was observed in the supernatant upon exposure of cells to potent ionic liquid (Figure 5a). ICP-AES data showed efflux of potassium and calcium when *C. albicans* 10231 cells were exposed to [C_16_MIM][Cl] (Figure 5b). Increased absorbance of supernatant and release of metal cations was a clear indication for leakage of intracellular contents during ionic liquid treatment.

### [C_16_MIM][Cl] Decreases Ergosterol Content

**FIGURE 6:**
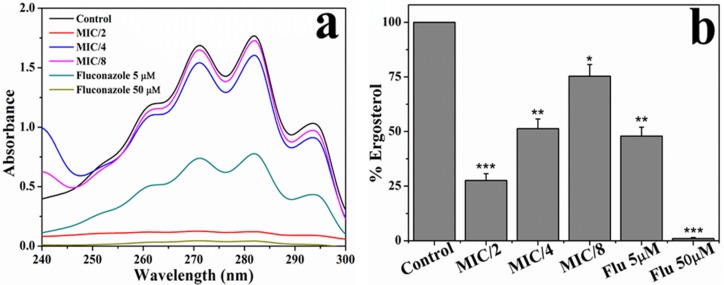
Effect of [C_16_MIM][Cl] on ergosterol content. **(a)** Absorption spectra of extracted sterols from *C. albicans* cells cultured in the presence of different concentrations of [C_16_MIM][Cl] and fluconazole. **(b)** Ergosterol content in *C. albicans* cells grown in the presence of [C_16_MIM][Cl] and fluconazole (*P < 0.05, **P < 0.01, ***P < 0.001, *n* = 2).

The effect of [C_16_MIM][Cl] on cell membrane ergosterol content was determined at sub MIC concentrations (MIC/2 to MIC/8). Absorption spectra of extracted sterols were shown in Figure 6a. A decrease in the absorbance of the sterols from 250 to 300 nm was evident when *C. albicans* 10231 were grown in the presence of [C_16_MIM][Cl]. The total sterol content, calculated from absorbance at 282 nm decreased in the cells cultured in the presence of sub MIC concentrations of [C_16_MIM][Cl]. Normalization to cell weight after ergosterol estimation and comparison with sterol content in control cells indicate the negative effect of ionic liquid on ergosterol content and the effect is concentration dependent (Figure 6b).

### [C_16_MIM][Cl] Induces ROS Production

**FIGURE 7:**
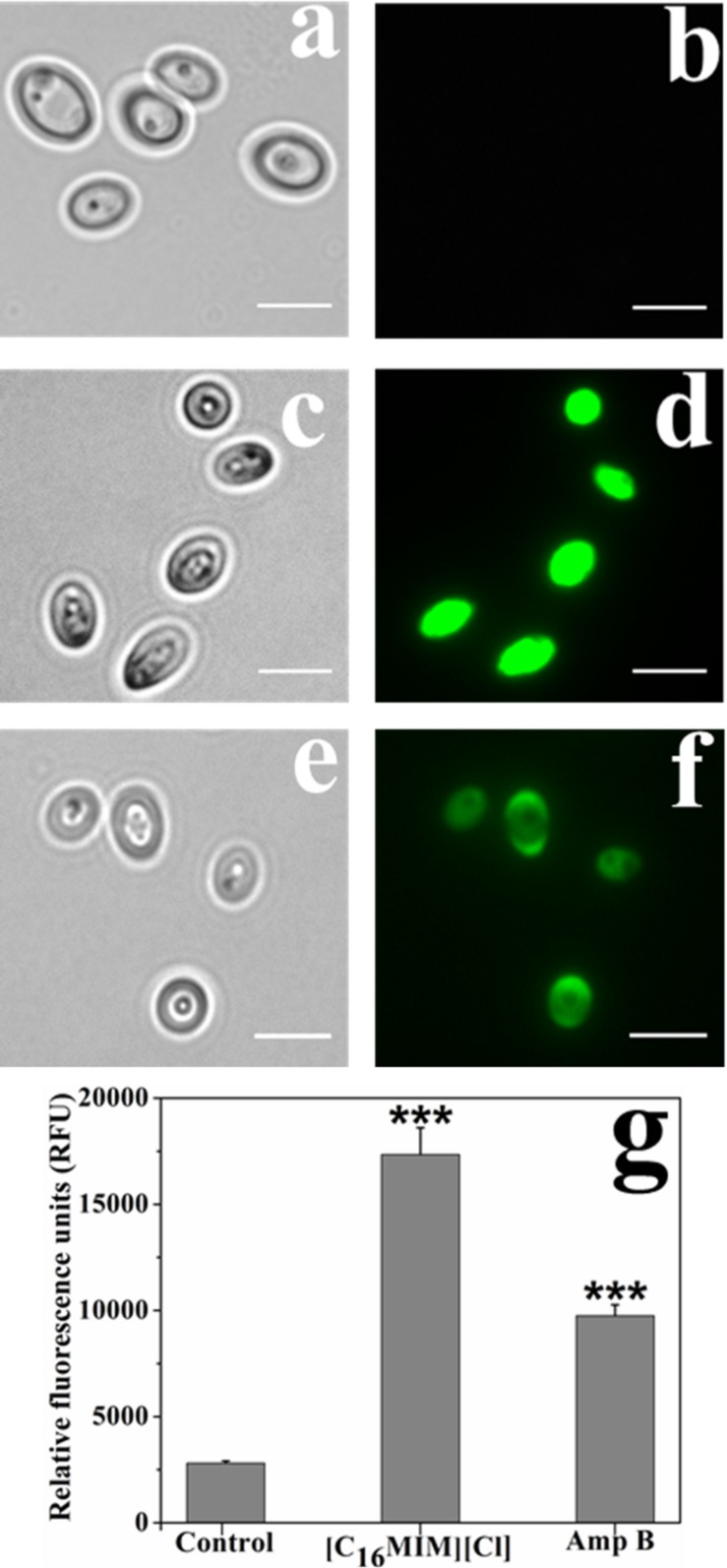
ROS in *C. albicans* cells before and after exposure to [C_16_MIM][Cl] and amphotericin B. Bright field and fluorescence images of control **(a,b)**, ionic liquid treated **(c,d)** and amphotericin B treated **(e,f)** cells. Scale bar = 5 μm. Quantitative estimation **(g)** of ROS in control, ionic liquid and amphotericin B treated cells. (Cells exposed to 10× MIC concentration for 3 h; ***P < 0.001, *n* = 4).

*Candida albicans* 10231 cells were exposed to [C_16_MIM][Cl] and amphotericin B and stained with DCFH-DA for determining ROS generation. ROS include superoxide anions, hydroxyl radicals, hydrogen peroxide, and singlet oxygen, which can oxidize DCFH-DA and generate DCF. Accumulation of DCF inside the cells is a direct estimation for ROS generation. Qualitative and quantitative measurement of ROS generation in *C. albicans* 10231 cells was shown in Figure 7. Fluorescence microscopic images revealed intense fluorescence from the *C. albicans* 10231 cells treated with [C_16_MIM][Cl] (Figure 7d). Cells treated with amphotericin B were also stained (Figure 7f), although to a lesser extent than ionic liquid treated cells. The fluorimetric data indicated significantly higher ROS in the *C. albicans* 10231 cells exposed to ionic liquid (Figure 7g). The ROS induced by amphotericin B were found to be significantly lower than that of ionic liquid treatment.

### [C_16_MIM][Cl] Decreases the Mitochondrial Membrane Potential and Inhibits Mitochondrial Activity

**FIGURE 8:**
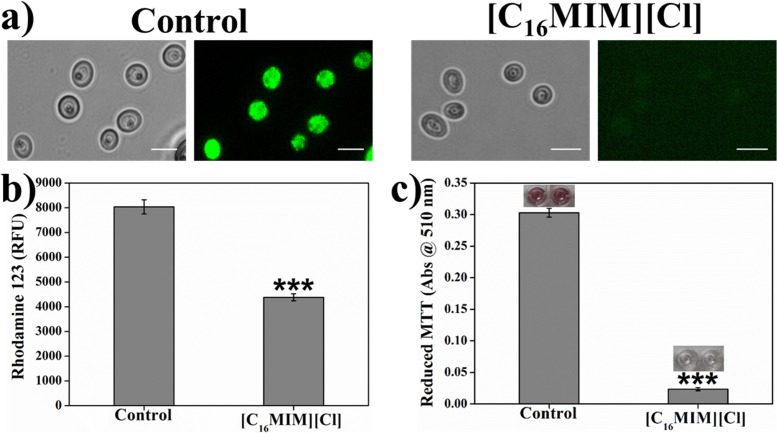
Effect of [C_16_MIM][Cl] on *C. albicans* mitochondrial membrane potential and mitochondrial activity. Cells exposed with [C_16_MIM][Cl] were incubated with Rhodamine 123 **(a,b)** and MTT **(c)**. Rhodamine 123 fluorescence from control and treated cells was observed under fluorescence microscope **(a)** and fluorescence was also quantified in multimode reader **(b)**. MTT reduction by control and treated cells is shown in figure **(c)** along with microtiter wells showing reduced MTT in control. Cells exposed to 10× MIC concentration for 3 h; ***P < 0.001, *n* = 4 for Rhodamine 123 assay, *n* = 2 for MTT assay.

Δψ_m_ is essential for mitochondrial energy metabolism for ATP synthesis (Zorova et al., 2018). Alterations in membrane potential can damage mitochondrial function and could affect the cell survival. Effect of [C_16_MIM][Cl] on Δψ_m_ was investigated by Rho123, a dye sequestered by active mitochondria. Hyperpolarization leads to increased accumulation of Rho123. But, depolarization results decreased accumulation of Rho123. Intense Rho123 staining was observed in control cells indicating the active Δψ_m_ (Figure 8a). Ionic liquid exposed *C. albicans* cells indicated faint staining with Rho123, indicating loss of membrane polarization (Figure 8a). Quantitative measurement of Rho123 fluorescence showed a significant decrease in ionic liquid exposed cells over control cells (Figure 8b). The results suggest that, ionic liquids cause loss of Δψ_m_ in *C. albicans* 10231 cells.

In addition to Δψ_m_, mitochondria function was also evaluated by estimating the activity of mitochondrial dehydrogenases. Metabolically active mitochondria can reduce colorless MTT to purple formazan which can be solubilized and measured calorimetrically. Failure of such reduction is an indication for the abnormal mitochondrial activity. MTT reduction to purple formazan was observed in control cells, indicating the normal activity of dehydrogenases. In case of ionic liquid exposed cells, formazan formation was severely impaired due to mitochondrial dysfunction (Figure 8c). The formazan development in control *C. albicans* 10231 cells inoculated in microtiter wells can be seen in the inset of Figure 8c.

## Discussion

The major challenges in developing new antifungal drugs or therapeutic strategies against *Candida albicans* are (i) its opportunistic pathogenicity causing both superficial and systemic fungal infections, (ii) its complex and polymorphic biofilm structure, and (iii) emergence of resistance in *C. albicans* strains against antifungal drugs like fluconazole. These challenges warrant the development of novel antifungal agents for effective treatment therapies. Ionic liquids have attracted attention for medical applications because of huge structural diversity (theoretically, 10^18^ compounds are possible) and tunability of structure which enable attaining desired biological activity and antimicrobial activities (Pendleton and Gilmore, 2015; Egorova et al., 2017). Among others, imidazolium ionic liquids are well studied for antimicrobial activities (Nancharaiah et al., 2012) and shown to possess antifungal activity (Table 1). For antibiofilm agents, it is desirable to have antimicrobial and surfactant activities (Choi et al., 2011). Imidazolium ionic liquids particularly with long-alkyl chains offer both these properties. In this study, we have chosen imidazolium ionic liquids with three different alkyl groups for determining (i) biofilm prevention and biofilm eradication activities on *C. albicans* strains and (ii) mode of action of promising ionic liquid on *C. albicans* cells. Although antifungal and antibiofilm activities of imidazolium ionic liquids have been studied previously (Bergamo et al., 2014, 2015; Dalla Lana et al., 2015; Ribas et al., 2016), their effects on preformed *C. albicans* biofilms and their molecular toxicity mechanisms are largely unknown. This study provided a clear insight into the biofilm eradication potential of alkylimidazolium ionic liquids and mechanism of action of prospective antifungal ionic liquid. Multi-marker approach was adopted for identifying targets and to discern the mode of action of promising antifungal ionic liquid.

### Antifungal, Antibiofilm and Biofilm Eradication Activity of Ionic Liquids

The antifungal, antibiofilm and biofilm eradication potential of three ionic liquids was determined *in vitro* against *C. albicans* strains. Alkylimidazolium ionic liquid with -butyl group was not inhibitory to *C. albicans* cells and considered as non-antifungal ionic liquid. Ionic liquids with -dodecyl and - hexadecyl groups were effective in preventing the growth of *C. albicans* and two other clinical strains, hence referred to as antifungal ionic liquids. This study re-confirmed that the antifungal activity of alkylimidazolium ionic liquids was dependent on the carbon chain length of alkyl group. The MIC and MFC values of ionic liquids were dependent on initial cell densities, that is, low concentrations were sufficient to inhibit the growth of 10^3^ cfu ml^–1^ than 10^6^ cfu ml^–1^. MIC values of potent antifungal ionic liquids were much lower than fluconazole against *C. albicans* 10231. However, the antifungal activity of two of the tested ionic liquids was comparable or slightly higher than amphotericin B. Biofilm formation by *C. albicans* 10231 and two clinical strains was effectively and completely inhibited in the presence of antifungal ionic liquids. The results of this study in terms of antifungal and antibiofilm activity of alkylimidazolium ionic liquids are in agreement with previous work on *C. albicans* (Schrekker et al., 2013; Bergamo et al., 2014). But, the effect of these antifungal ionic liquids on preformed biofilms is largely unknown (Table 1). The effect of antifungal drugs on adherent cells or biofilms is of clinical relevance because (i) biofilm formation often precedes treatment and (ii) biofilms are much more resistant to antifungals. In this context, the biofilm eradication potential of antifungal ionic liquids was determined for the first time in terms of viability loss and dispersal of preformed *C. albicans* biofilms. Antifungal ionic liquid with -hexadecyl group was able to effectively disperse 24 h old *C. albicans* biofilm (Figure 2c). Interestingly, antifungal ionic liquid with –dodecyl group was not effective in dispersing the biofilm even at higher concentrations (Figure 2b and Supplementary Figures S5C,D). But, this antifungal ionic liquid remarkably decreased the viability and metabolic activity of *C. albicans* cells in the preformed biofilm (Figure 2d). The biofilm eradication potential results suggest that antifungal ionic liquids are suitable for treating *C. albicans* infections.

### Mechanism of Action of Antifungal Ionic Liquids

The microscopic observation revealed that the cells exposed to [C_16_MIM][Cl] were smaller than the untreated control cells (Figure 3). A significant reduction in cell volume was noticed in *C. albicans* 10231 cells exposed to antifungal ionic liquid for few hours. Shrinkage of *C. albicans* cells was observed upon exposure to antifungal agents such as apigenin, silver nanoparticles (Li et al., 2013; Lee et al., 2018). The cell shrinkage was often associated with cell membrane permeabilization and leakage of intracellular contents (Lee et al., 2018). Ionic liquid induced cell membrane permeabilization was already reported in bacteria and microalgae (Nancharaiah et al., 2012; Reddy et al., 2017). An increased absorbance (at 260 nm) and metal ions (K^+^ and Ca^2+^) in the water surrounding *C. albicans* 10231 during exposure to antifungal ionic liquid suggested cell membrane permeabilization or damage (Figure 5). Membrane permeabilization was indeed confirmed in ionic liquid treated *C. albicans* 10231 cells by the preferential uptake of PI but not by control cells (Figure 4). The intracellular concentrations of alkali metal ions (i.e., K^+^, Na^+^) are important for maintaining cell volume, pH and cell membrane potential in yeasts (Arino et al., 2010). Therefore, leakage of metal cations (K^+^ and Ca^2+^) and other intracellular material (UV absorbing substances) are responsible for reduction in cell volume. Ergosterol, another important constituent of fungal membranes was quantified in *C. albicans* 10231 cells cultured in the presence of [C_16_MIM][Cl]. Ergosterol is a major sterol in the fungal cell membrane and adequate levels are essential for maintaining membrane integrity and other membrane functions (Hu et al., 2017). Hence, ergosterol and its biosynthetic pathways are important targets in the development of antifungal agents (Onyewu et al., 2003). A significant decrease in ergosterol content of *C. albicans* 10231 cells cultured in the presence of antifungal ionic liquid (Figure 6) suggests possible inhibition of ergosterol biosynthesis. This was in agreement with the observations of Schrekker et al. (2013) that imidazolium ionic liquids interfere in the ergosterol biosynthesis and cause a reduction in its levels in the cell membrane. It was hypothesized that imidazolium ionic liquids interrupt conversion of lanosterol to ergosterol by inhibiting lanosterol 14α-demethylase (Schrekker et al., 2013). However, this is yet to be validated experimentally.

Generation and accumulation of intracellular ROS was prominent in *C. albicans* 10231 cells treated with [C_16_MIM][Cl] (Figure 7). In yeasts, ROS are majorly produced in the mitochondria (Knorre et al., 2016). High ROS levels are detrimental as they cause oxidative damage to intracellular molecules and cell membrane lipids (Sun et al., 2017). Interestingly, ROS production is one of the mechanisms by which yeast cells senses mitochondrial dysfunction (Knorre et al., 2016). The mitochondrial dysfunction in *C. albicans* 10231 cells was assessed by Δψ_m_ and dehydrogenases activity. Rho123, a cationic and lipophilic dye used for quantifying Δψ_m_ because this dye can specifically stain negatively charged mitochondria (Dananjaya et al., 2017). A significant decrease in Rho123 staining by *C. albicans* 10231 upon treatment with antifungal ionic liquid (Figure 8) indicated disruption of membrane potential. MTT assay indicated almost complete loss of dehydrogenases activity asserting mitochondrial dysfunction in ionic liquid treated *C. albicans* 10231 cells. Cell volume reduction, intracellular ROS production and mitochondrial dysfunction are often observed in apoptosis (Pereira et al., 2008). Therefore, future experimental work should investigate mode of action of antifungal ionic liquids in this direction.

### Clinical Relevance

Fungal infections alone account for approximately 11.5 million life-threatening infections and 1.6 million deaths annually around the globe (Fuentefria et al., 2018; Almeida et al., 2019). Treatment of fungal infections is a challenge in clinical settings because of limited number of antifungal drugs for treating invasive infections, inefficacy in preventing infections, difficulty in administering and combination of all these factors (Desai et al., 2014; Beardsley et al., 2018). This is amplified by emerging antifungal resistance and resistance conferred by fungal biofilms. Ionic liquids, unique class of compounds, are seen as promising assets for treating life-threatening fungal infections due to their structural diversity and tunable physical and chemical properties which contribute to the synthesis of a large number of compounds (Hartmann et al., 2016). Imidazolium ionic liquids are promising because of their strong antifungal and biofilm inhibition activities. The other important attributes of these compounds are (i) broad spectrum activity on bacteria and fungi (Nancharaiah et al., 2012; Dalla Lana et al., 2015; Bergamo et al., 2016) and (ii) multiple cellular targets for exhibiting antifungal activity as demonstrated in this study. Antifungal compounds with multiple pharmaceutical targets are promising for evading resistance development, a menace in antifungal therapy. Antifungal susceptibility testing and other *in vitro* assays can screen a large number of compounds, identify effective compounds and identify cellular targets. Effective ionic liquids are suitable for prospective applications in antifungal creams for topical applications, disinfection of surgical tools and treatment of dental lines in hospital settings. The results of *in vitro* assays are useful for guiding treatment choices (Beardsley et al., 2018) and complementation with *in vivo* studies is necessary for considering potential clinical applications.

Schrekker et al. (2016) evaluated the biocompatibility of imidazolium ionic liquids *in vitro* using L929 fibroblast cells of mice. These tests revealed that cytotoxicity increases with an increase in alkyl chain length from –butyl to –decyl and –hexadecyl group. 1-butyl-3-methylimidazolium chloride was fully biocompatible with no toxicity on fibroblast cells. Whereas, 1-decyl-3-methylimidazolium chloride and 1-hexadecyl-3-methylimidazolium chloride were cytotoxic to fibroblast cells, respectively, at 50 to 500 μg ml^–1^ and 10 to 500 μg ml^–1^. Thus, biocompatibility of –hexadecyl containing ionic liquid was reported to be good at <10 μg ml^–1^ with minimal toxicity on fibroblast cells suggesting that low concentrations of these compounds are still safe. Cytotoxicity data for 1-dodecyl-3-methylimidazolium iodide is not available, although it can be predicted to possess the toxicity between –decyl and –hexadecyl alkyl group containing imidazolium ionic liquids. The concentrations of 1-hexaydecyl-3-methylimidazolium chloride at 0.84 μg ml^–1^ and 1.69 μg ml^–1^ for antifungal and antibiofilm (for complete biofilm prevention) activities, respectively, are below the reported toxicity value of 10 μg ml^–1^ on fibroblast cells (Schrekker et al., 2016) and seems to be safe. Nevertheless, additional biocompatibility studies of antifungal ionic liquids are warranted for considering these compounds in prospective formulations. Therefore, further research should focus on (i) efficacy, (ii) formulation and co-administration with other compounds, and (iii) cellular toxicity using *in vivo* models.

## Data Availability Statement

All datasets generated for this study are included in the article/Supplementary Material.

## Author Contributions

GK and YN conceived and designed the experiments. GK performed the experiments and processed the data. GK and YN interpreted the data. GK and YN wrote the manuscript.

## Conflict of Interest

The authors declare that the research was conducted in the absence of any commercial or financial relationships that could be construed as a potential conflict of interest.

## Footnotes

1 http://www.cdc.gov/fungal/diseases/candidiasis/
