# Supplementary material for: Alkylimidazolium Ionic Liquids as Antifungal Alternatives: Antibiofilm Activity Against Candida albicans and Underlying Mechanism of Action
Source: Front Microbiol. 2020 Apr 21;11:730. doi: 10.3389/fmicb.2020.00730 (PMC7186398; doi:10.3389/fmicb.2020.00730)
Supplement: Supplementary file 1 [file Data_Sheet_1.docx]

**Supplementary Information**

**Alkylimidazolium ionic liquids as antifungal alternatives: Antibiofilm activity against *Candida albicans* and underlying mechanism of action**

G. Kiran Kumar Reddy^a,b^, Y.V. Nancharaiah^a,b,*^

^a^Biofouling and Biofilm Processes, Water and Steam Chemistry Division, Chemistry Group, Bhabha Atomic Research Centre, Kalpakkam - 603102, India.

^b^Homi Bhabha National Institute, Anushakti Nagar Complex, Mumbai - 400 094, India.

*Corresponding author:

Y.V. Nancharaiah,

Biofouling and Biofilm Processes,

Water and Steam Chemistry Division,

Bhabha Atomic Research Centre,

Kalpakkam - 603102, Tamil Nadu, India.

Email: yvn@igcar.gov.in, venkatany@gmail.com.

Tel:+91 44 27480203, Fax; +9144 27480097

**Total pages: 7**

**Number of tables: Nil**

**Number of figures: 7**

| 1-butyl-3-methylimidazolium chloride  [C_4_MIM][Cl] | **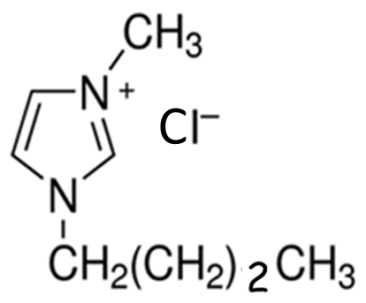** |
| --- | --- |
| 1-dodecyl-3-methylimidazolium iodide  [C_12_MIM][I] | **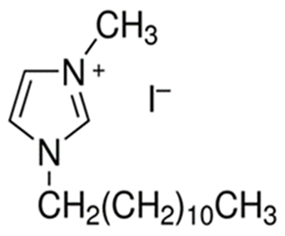** |
| 1-hexadecyl-3-methylimidazolium chloride  [C_16_MIM][Cl] | **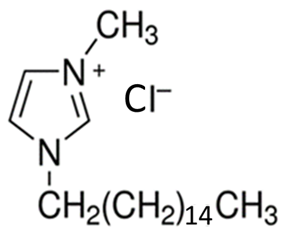** |

Figure S1. Chemical structure of imidazolium ionic liquid compounds used in this study.


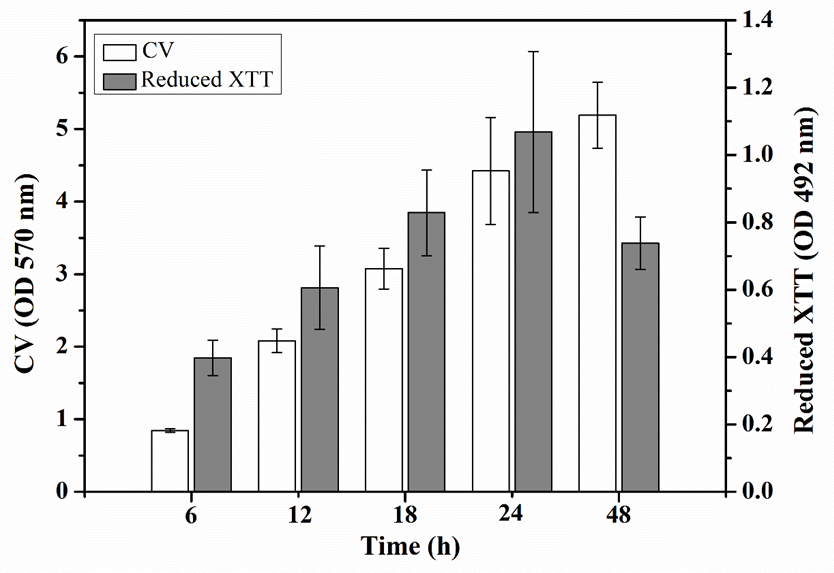


Figure S2. Biofilm development by *C. albicans* ATCC 10231 at different time intervals. Biofilm biomass and metabolic activity was quantified using crystal violet (CV) and XTT, respectively.

Figure S3. Biofilm formation after a 3 h adhesion step by*C. albicans* 10231 at different concentrations of [C_4_MIM][Cl] (a), [C_12_MIM][I] (b), and [C_16_MIM][Cl] (c).

Figure S4. Planktonic growth (OD600) and biofilm formation (Crystal violet, OD570) in presence of higher concentrations (500 and 1000 µM) of ionic liquid anions (Cl^-^ and I^-^) and 1-methylimidazole.

Figure S5. Image of 96 well microtiter plate showing the eradication of preformed *C. albicans* ATCC 10231 biofilms after challenging with different concentrations of ionic liquids.


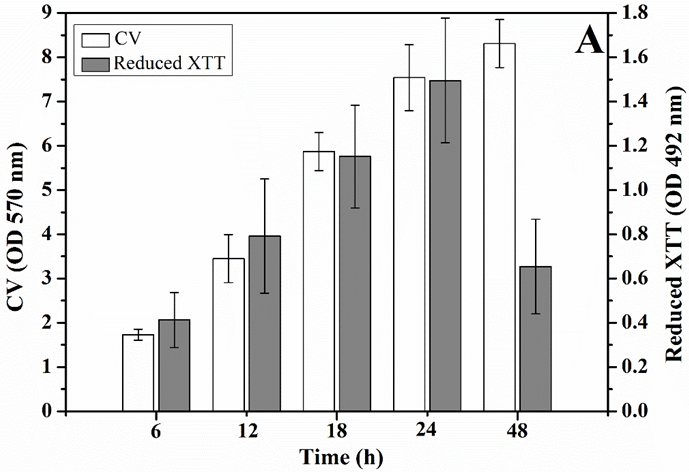


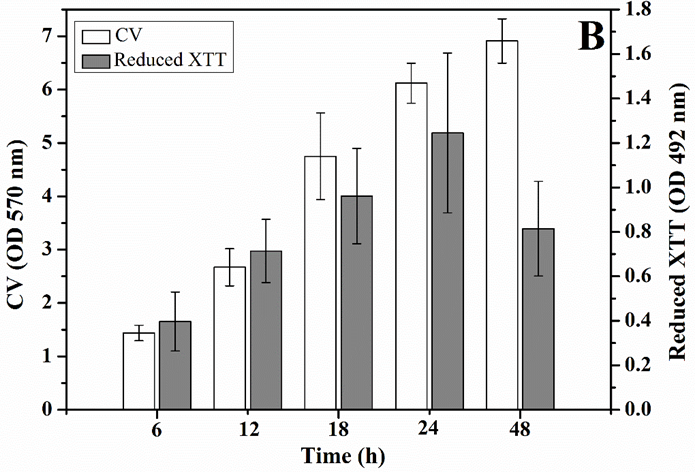


Figure S6. Biofilm development by *C. albicans* clinical isolates, CA i16 (A) and CAi21 (B) at different time intervals. Biofilm biomass and metabolic activitywere quantified using crystal violet (CV)staining and XTT reduction, respectively.

Figure S7. Antibiofilm (a, b) and biofilm eradication potential (c, d) of antifungal alkylimidazolium ionic liquids on fluconazole resistant clinical *C. albicans* isolates (CA i16 and CA i21).
